# Supplementary material for: Phosphoproteomic Analysis Identifies TYRO3 as a Mediator of Sunitinib Resistance in Metastatic Thymomas
Source: Cancers (Basel). 2022 Sep 29;14(19):4762. doi: 10.3390/cancers14194762 (PMC9562918; doi:10.3390/cancers14194762)
Supplement: Supplementary file 1 [file cancers-14-04762-s001.zip › cancers-1932089-supplementary/cancers-1932089-supplementary.pdf]

cancers MDPI

Supplementary

Phosphoproteomic analysis identifies TYRO3 as a mediator of sunitinib resistance in metastatic thymomas

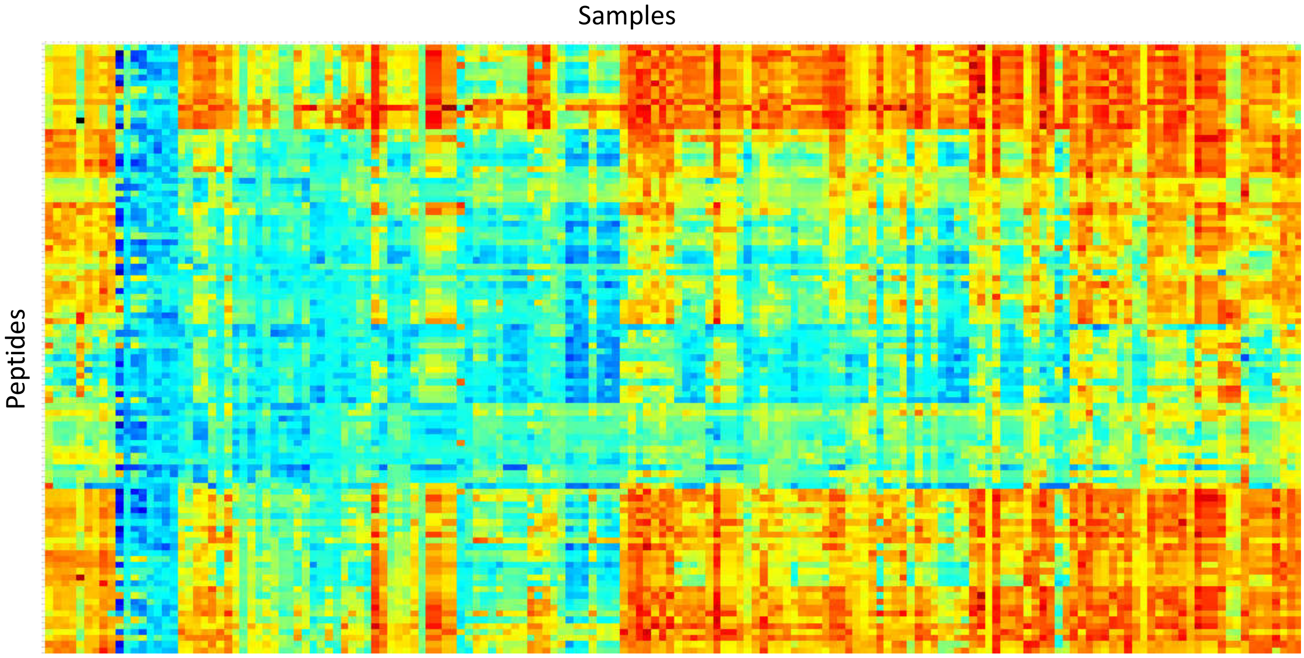

Figure S1. Peptide signal raw data map. Raw peptide signals of all measured samples generated in the Bionavigator software (PamGen International B.V., 's-Hertogenbosch, The Netherlands). From 144 peptide signal the median signal minus background was calculated. Red = high signal value, blue = low signal value.

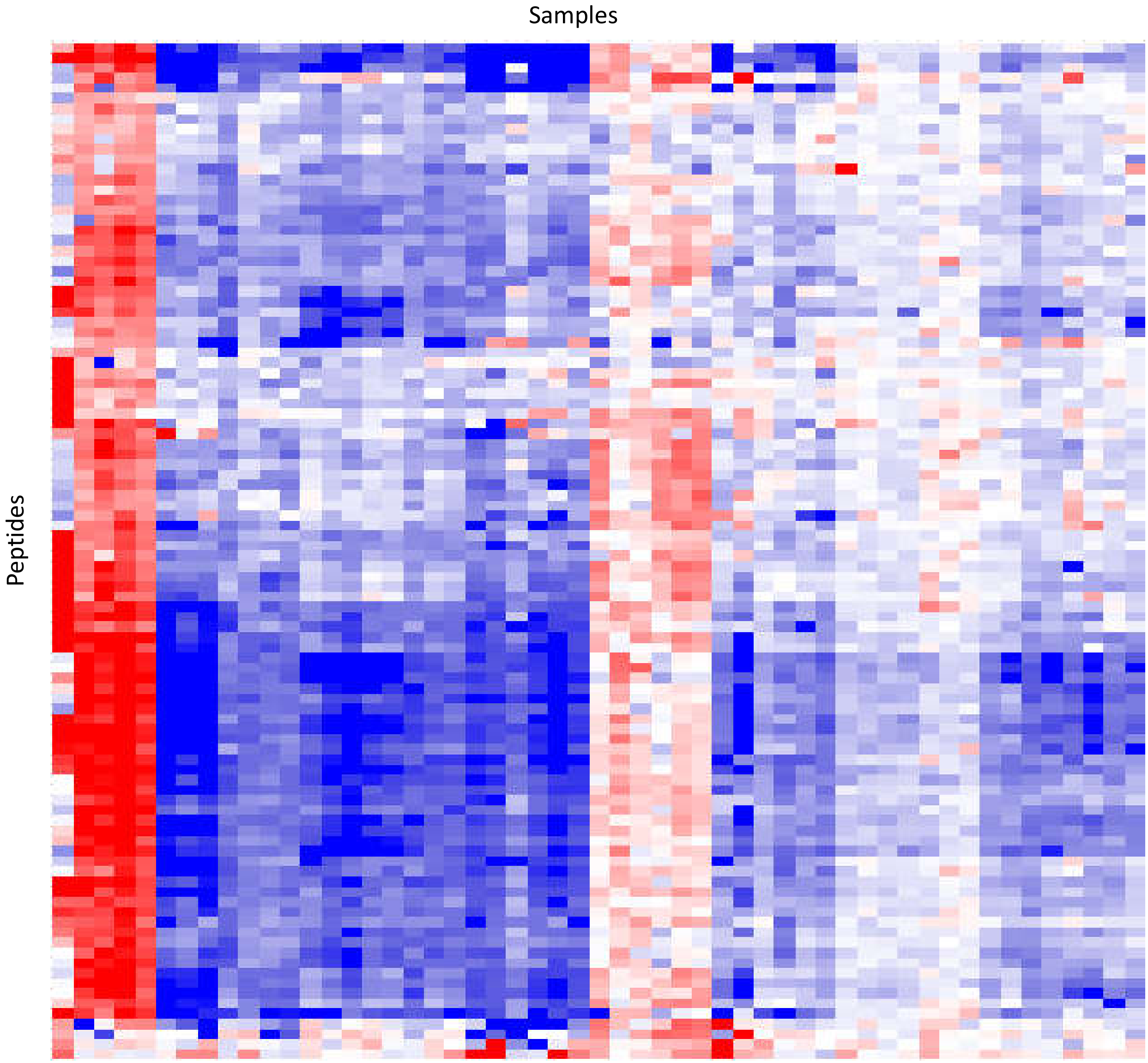

Figure S2. Peptide signal ratio map. To stabilize the variance of the overall kinase activity due to different sample ages, the ratio of the sunitinib treated vs. the DMSO control peptide signal was generated. Red = high ratio, blue = low ratio.

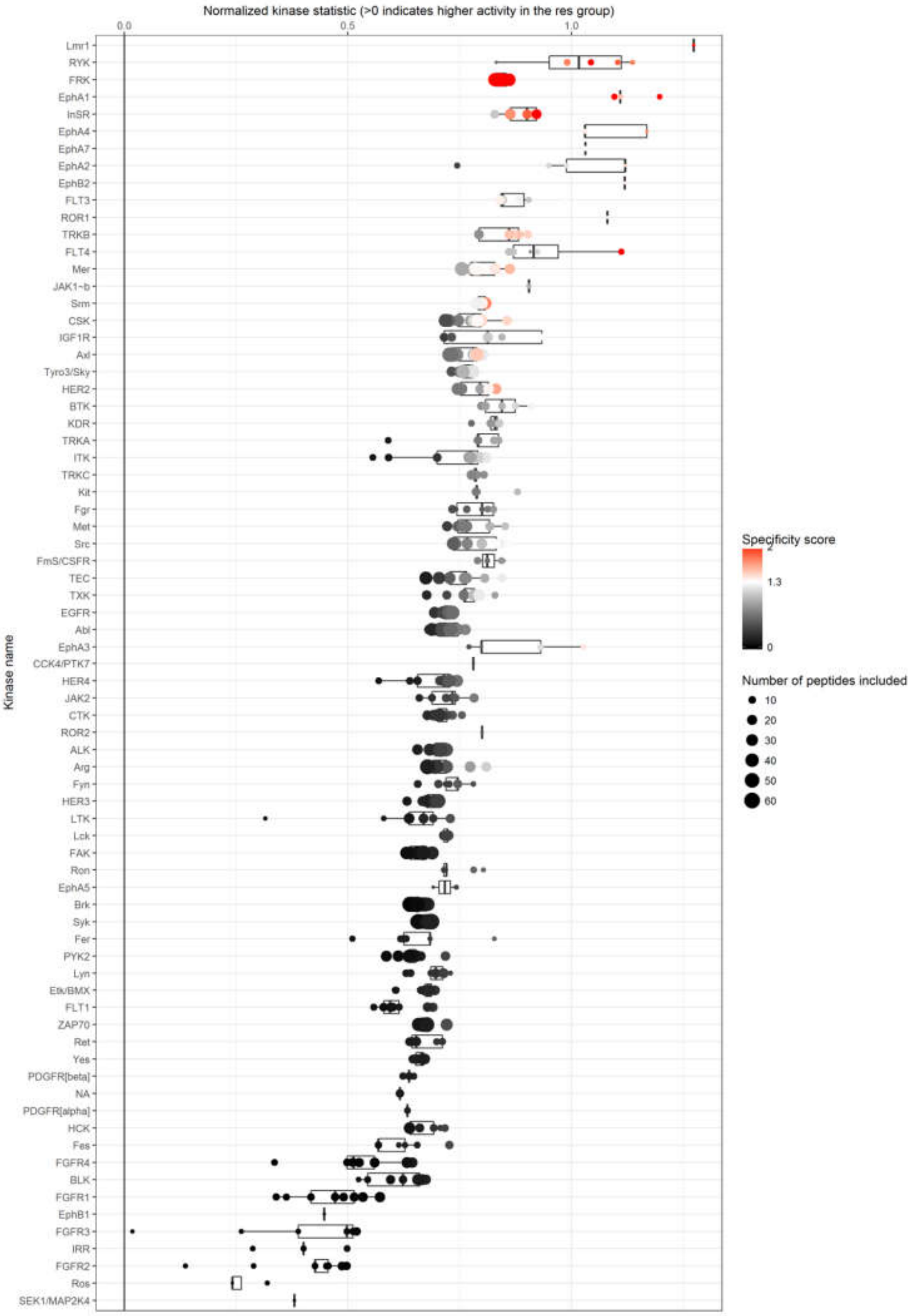

Figure S3. All predicted upstream active RTKs in cell lines. The value on the x-axis indicates the activity change of each kinase relative to untreated control. The colour indicates the reliability of the prediction (specificity score). The dot size indicates the number of phosphorylated peptides on which the prediction was based.

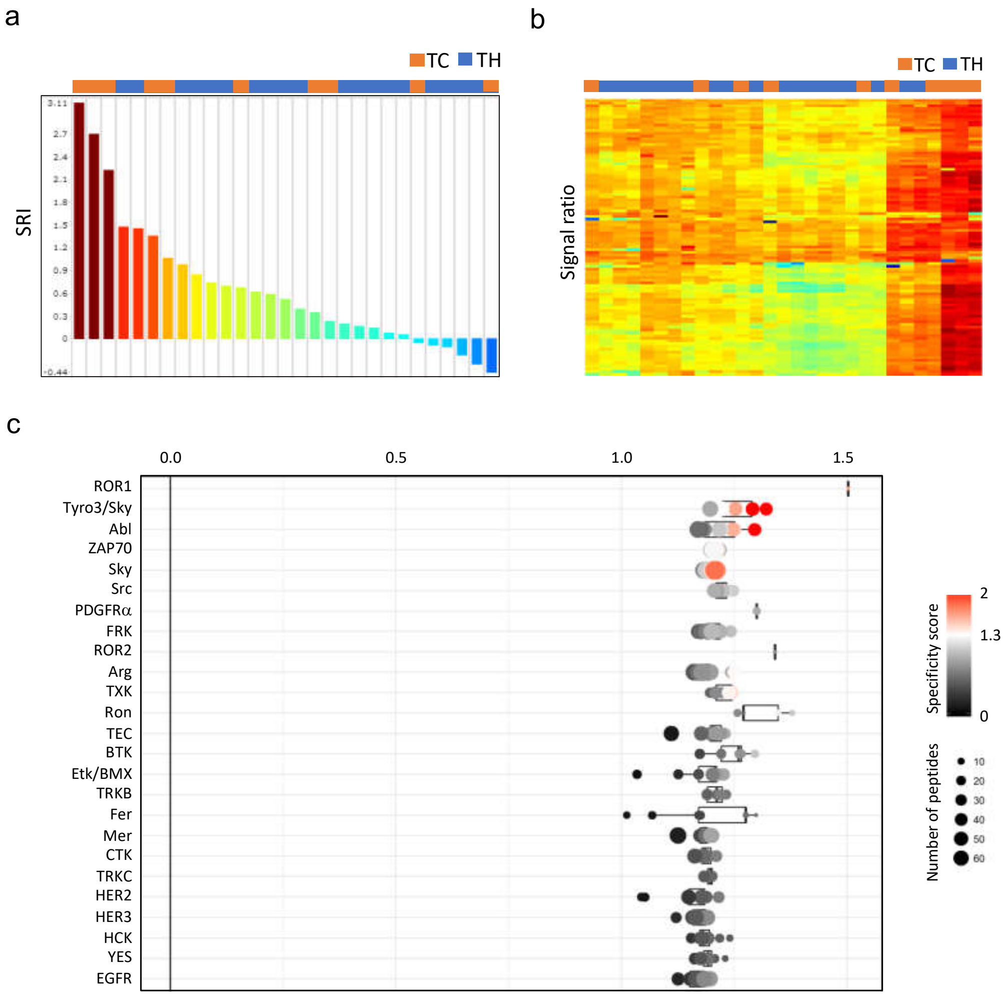

Figure S4. Prediction of sunitinib response based on SRI in TH and TC tissue samples and prediction of upstream RTKs across all samples. a) Waterfall plot of sunitinib response of all TET predicted by SRI. b) Unsupervised hierarchical clustering of of phosphorylation ratios in TET samples (with/without sunitinib spike-in). c) Prediction of involved upstream tyrosine kinases (top 25, ranked by relative change). The value on the x-axis indicates the activity change of each kinase relative to untreated control. The colour indicates the reliability of the prediction (specificity score). The dot size indicates the number of phosphorylated peptides on which the prediction was based.

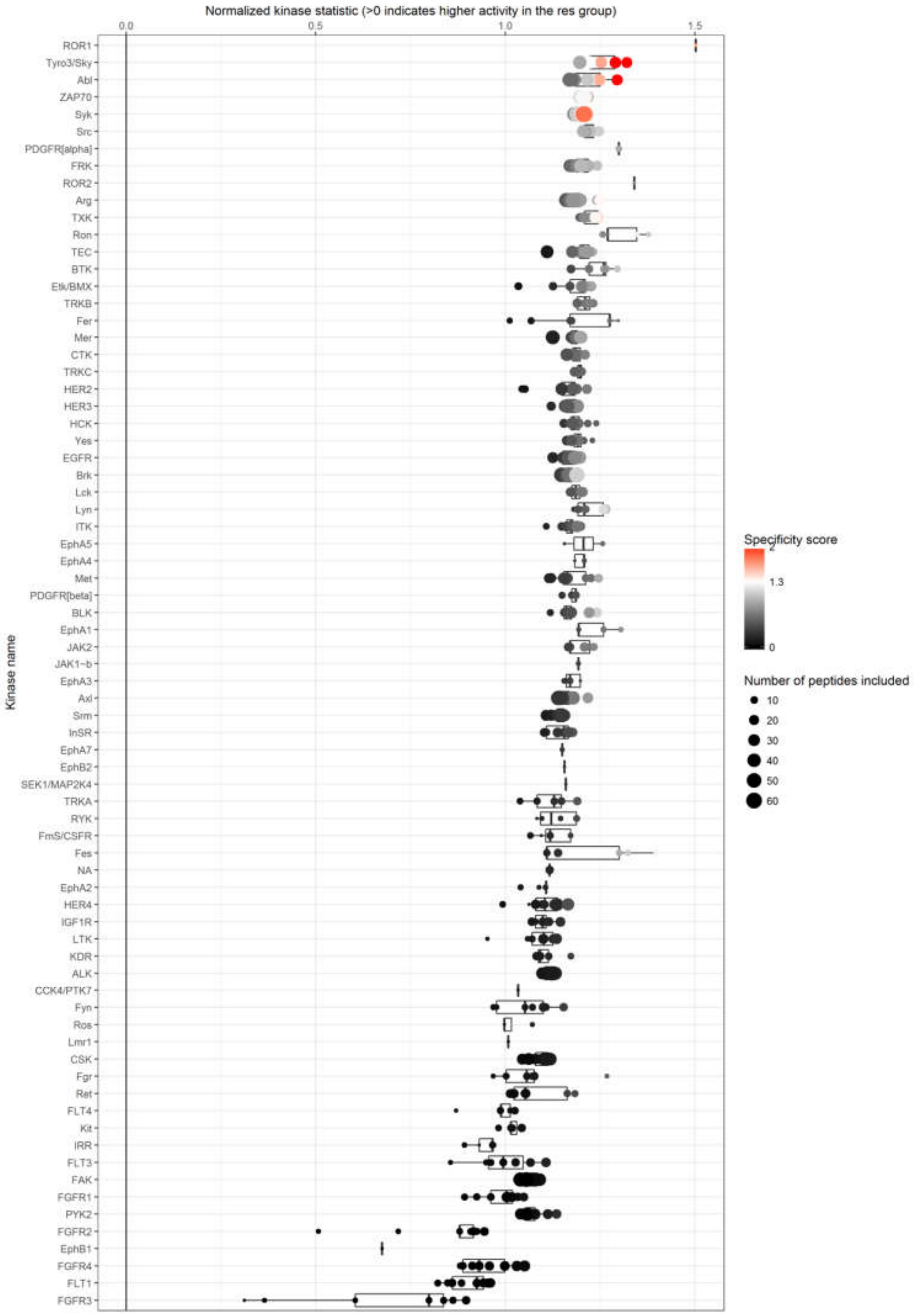

Figure S5. All predicted active upstream RTKs in TH and TC. The value on the x-axis indicates the activity change of each kinase relative to untreated control. The colour indicates the reliability of the prediction (specificity score). The dot size indicates the number of phosphorylated peptides on which the prediction was based.

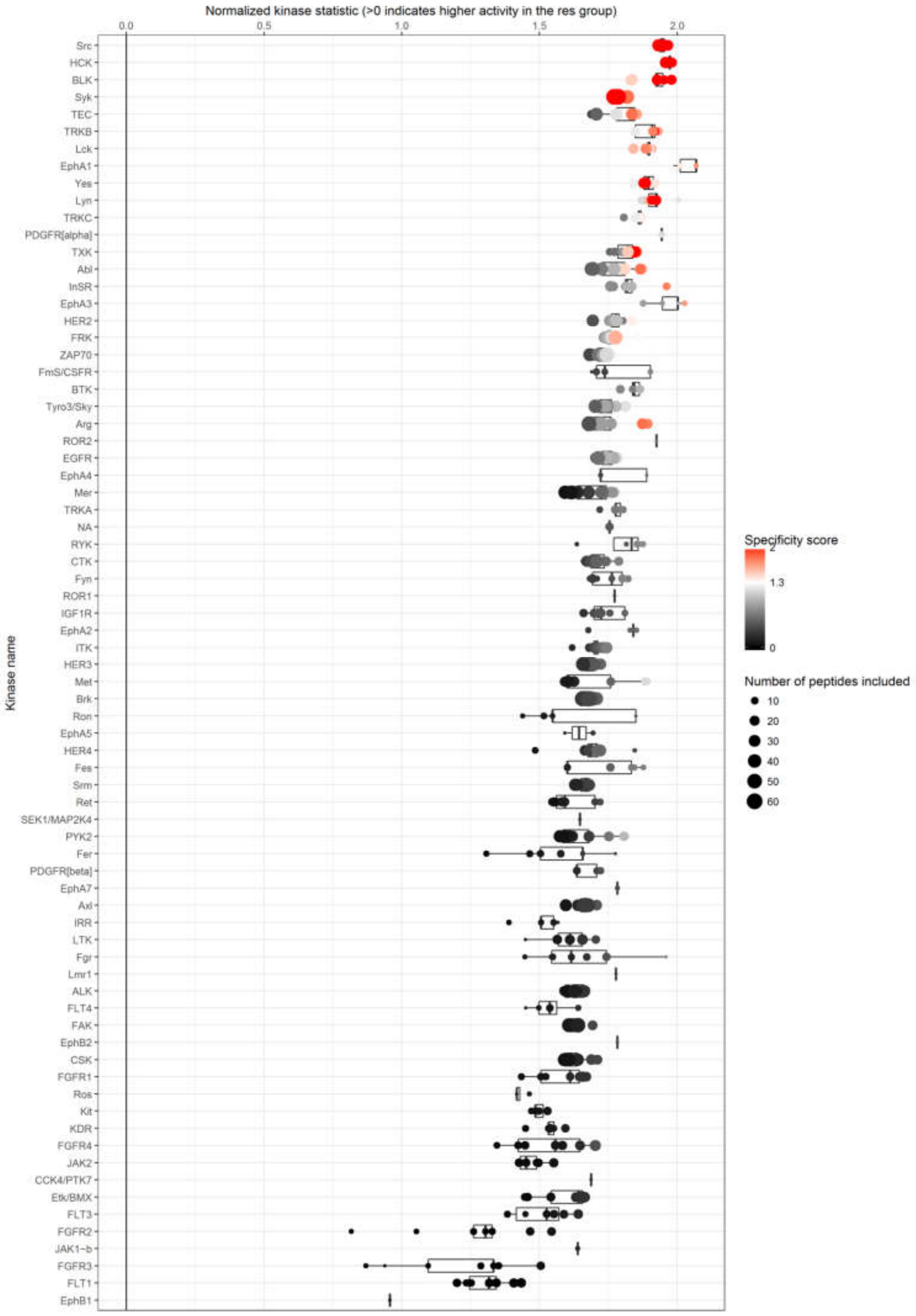

Figure S6. All predicted active upstream RTKs activated in resistant in TC. The value on the x-axis indicates the activity change of each kinase relative to untreated control. The colour indicates the reliability of the prediction (specificity score). The dot size indicates the number of phosphorylated peptides on which the prediction was based.

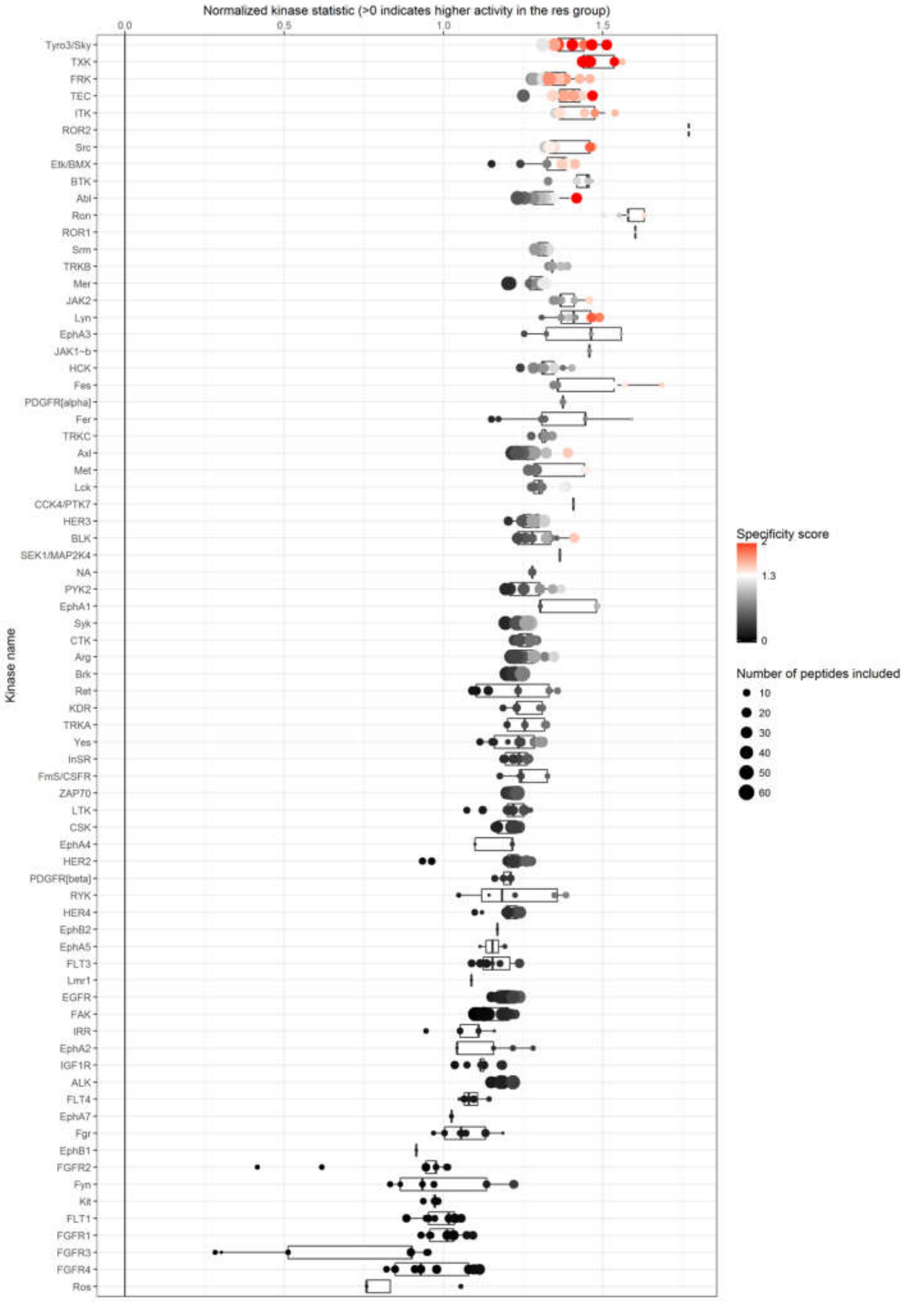

Figure S7. All predicted active upstream RTKs in resistant in TH. The value on the x-axis indicates the activity change of each kinase relative to untreated control. The colour indicates the reliability of the prediction (specificity score). The dot size indicates the number of phosphorylated peptides on which the prediction was based.
