# Supplementary figures and images for: Phosphoproteomic Analysis Identifies TYRO3 as a Mediator of Sunitinib Resistance in Metastatic Thymomas
Source: Cancers (Basel). 2022 Sep 29;14(19):4762. doi: 10.3390/cancers14194762 (PMC9562918; doi:10.3390/cancers14194762)

Unprocessed Figure 4a

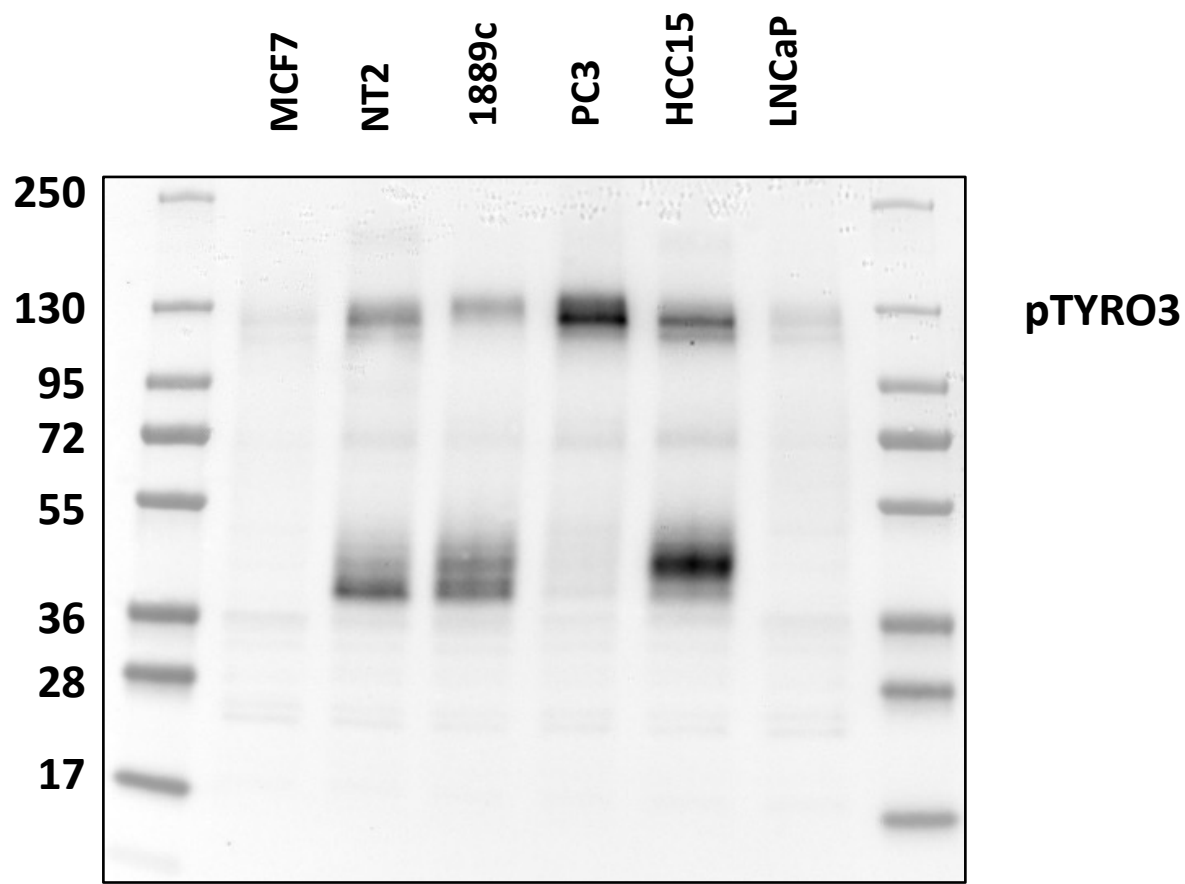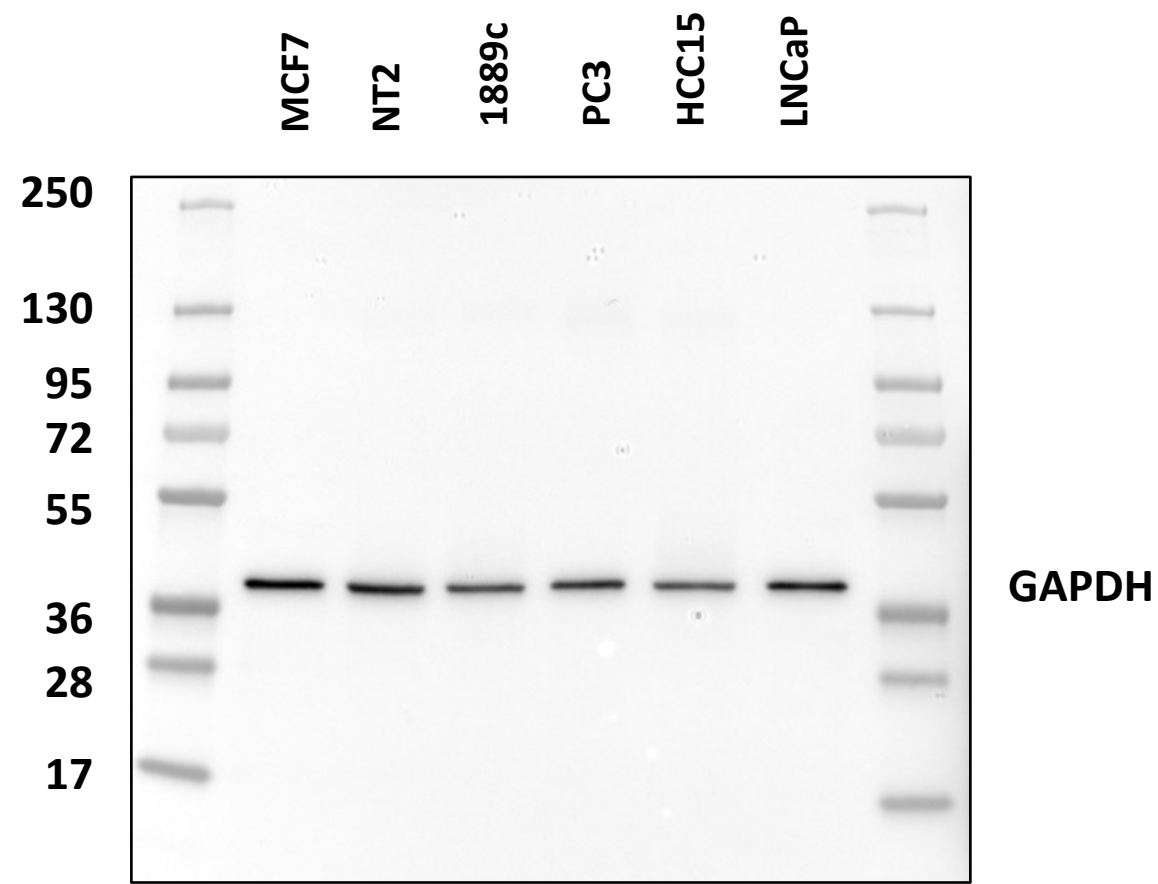

Unprocessed Figure 4d

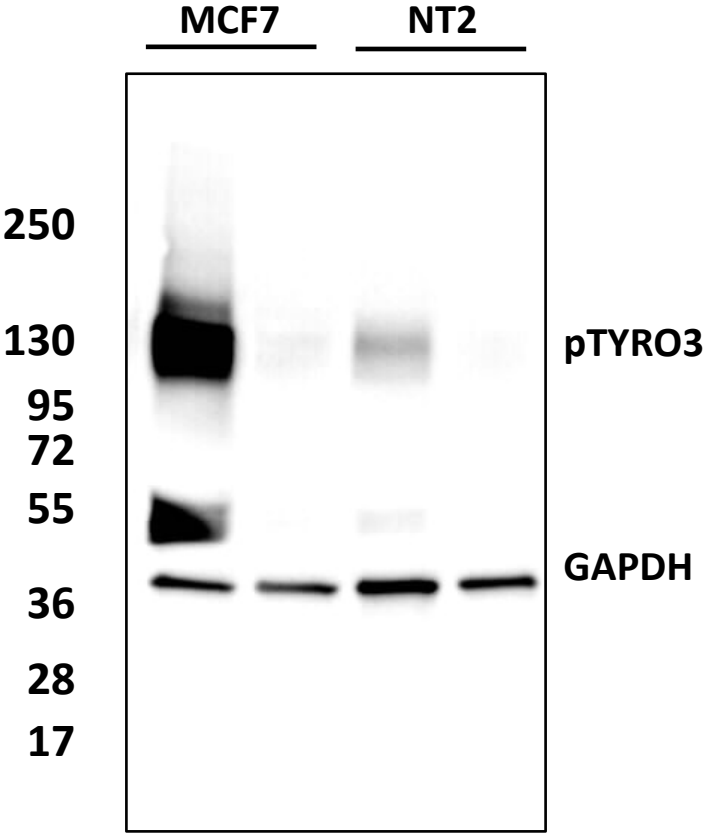

Unprocessed Figure 4e

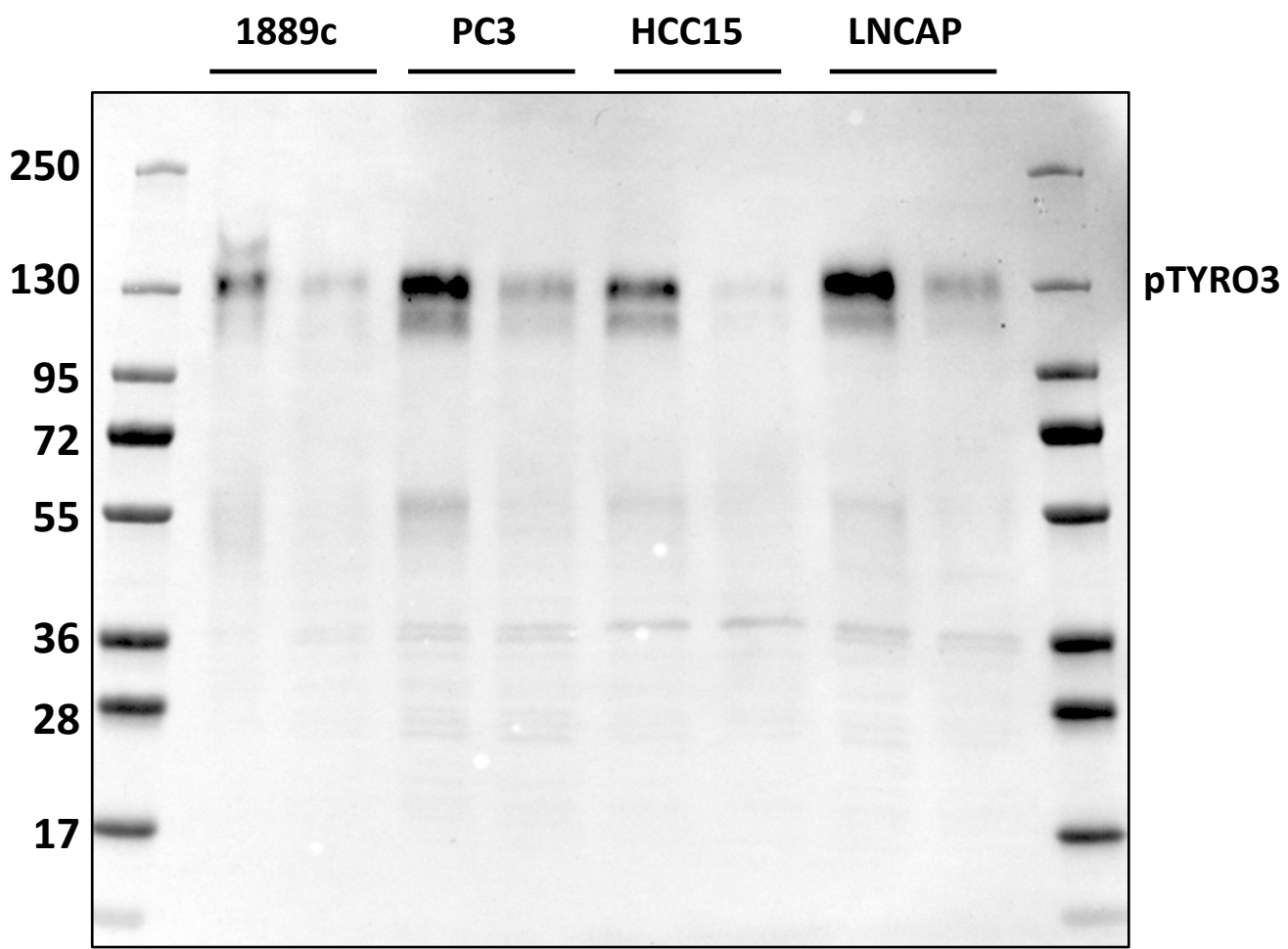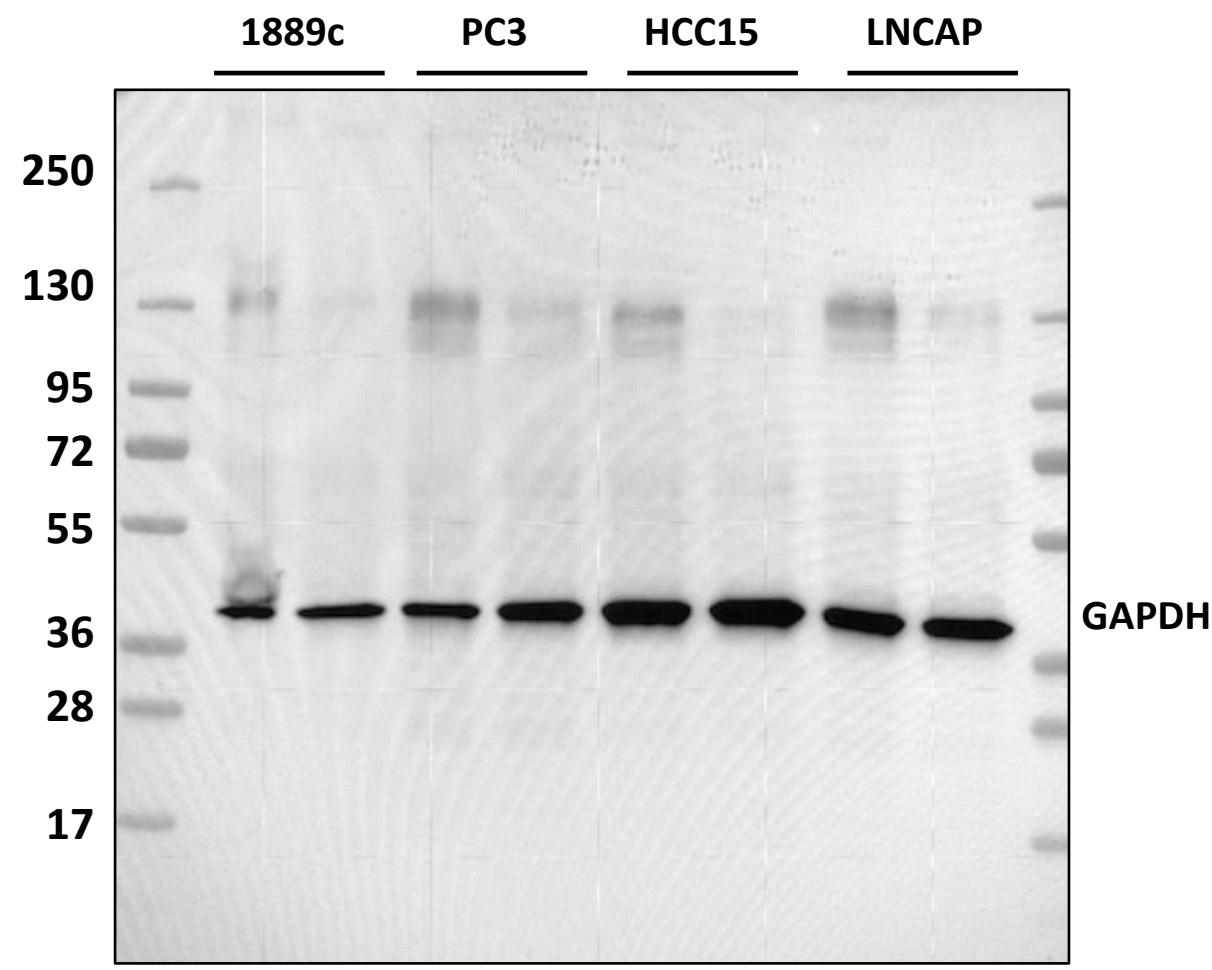

Supplement: Supplementary file 1 [file cancers-14-04762-s001.zip › cancers-1932089-supplementary/cancers-1932089-Figure S8.pdf]
